# Association between total bilirubin/Albumin ratio and all-cause mortality in acute kidney injury patients: A retrospective cohort study

**DOI:** 10.1371/journal.pone.0287485

**Published:** 2023-11-01

**Authors:** Ximei Huang, Yunhua Huang, Min Chen, Lin Liao, Faquan Lin

**Affiliations:** Key Laboratory of Clinical Laboratory Medicine of Guangxi Department of Education, Department of Clinical Laboratory, The First Affiliated Hospital of Guangxi Medical University, Guangxi Zhuang Autonomous Region, Nanning, China; University of Colombo Faculty of Medicine, SRI LANKA

## Abstract

**Background:**

The association between the total bilirubin/albumin (B/A) and the all-cause mortality of critically ill patients with acute kidney injury (AKI) remains unclear. This retrospective study aimed to investigate the relationship between B/A ratio and mortality in patients with AKI.

**Methods:**

The clinical data of AKI patients in the Medical Information Mart for Intensive Care III (MIMIC-III) database were retrospectively analyzed. Patients were divided into the low and high B/A groups (B/A ≤ 0.25 and B/A > 0.25, respectively). The primary outcome was 28-day all-cause mortality, and the secondary outcomes were 60-day, 1-year and 4-year all-cause mortality. Kaplan–Meier survival curves and Cox proportional risk models were constructed to evaluate the effect of B/A on survival outcomes.

**Results:**

The 28-day mortality rates were 18.00% and 25.10% in the low and high B/A groups, respectively (P < 0.001). The Kaplan–Meier analysis showed that patients with higher B/A values had higher all-cause mortality risk (log-rank P < 0.0001). The multivariate Cox proportional risk analysis showed that B/A was an independent risk predictor for death at 28 days, 60 days, 1 year, and 4 years.

**Conclusion:**

B/A is an independent risk factor for increased mortality in patients with AKI and may be used as a predictor of clinical outcomes in AKI.

## Introduction

Acute kidney injury (AKI) is a clinical syndrome in which the excretory function of the kidney decreases rapidly (hours to days), decreasing the glomerular filtration rate and increasing serum creatinine [1]. AKI is increasingly common in developed and developing countries, with high morbidity and mortality rates [2]. Epidemiological surveys have revealed that approximately 15,000 per 1 million people in developed countries experience AKI, and this number is increasing [3]. In addition, studies have shown that approximately 12.2% of hospitalized patients [4] and 57.3% of ICU patients [5] have AKI. ICU patients are also at a significantly increased risk of death from AKI during hospital admission; The mortality rate of AKI patients in the ICU is higher than the average ICU patient mortality rate [6]. These high morbidity and mortality rates place a substantial burden on global health and increase the economic burden of the population [7]. Scoring systems and constructed models are available for predicting the prognosis of AKI patients and can provide information that helps prevent and treat AKI. However, none of the scoring systems currently used to predict the illness severity and prognosis of critically ill patients are specific to AKI patients [8]. Second, some of the constructed prediction models have poor performance, and no accurate model is available [9]. Therefore, there is a need to select appropriate biological indicators to predict AKI patient prognosis to achieve adequate prevention and treatment.

Serum total bilirubin and albumin concentrations are associated with AKI development and clinical outcomes. High levels of total serum bilirubin lead to a decrease in arterial pulse pressure and intraglomerular pressure, which has a direct toxic effect on the renal tubules [10]. The incidence of AKI in patients with serum total bilirubin >2.0 mg/dl has been reported to be 16.4%; serum total bilirubin >2.0 mg/dl can promote AKI progression [11]. Previous studies have shown that elevated total serum bilirubin is a risk factor for AKI development (OR = 1.06, 95% CI: 0.60–1.80, P<0.05) [12]. Albumin is a key protein with multiple functions, including osmolarity regulation, antioxidant activity and anti-inflammatory effects [13]. Studies have shown that hypoalbuminemia in critically ill patients is associated with AKI development [14]. Other studies have shown that hypoalbuminemia is an important independent predictor of death in AKI patients, and monitoring serum albumin can help predict the risk of death in AKI patients [15].

It has been reported that the total bilirubin/albumin ratio (B/A) is associated with poor prognosis and mortality in critically ill patients [16]; however, there are few studies on its relationship with AKI patient prognosis. Therefore, we retrospectively analyzed the relationship between B/A and mortality in patients with AKI to provide a reference for prognostic monitoring and clinical treatment aspects.

## Methods

### Data source and collection

The study data were extracted from the MIMIC–III database, which contains information on patient hospitalizations at the Higher Medical Center in Boston, Massachusetts, USA, from 2001 to 2012. The database has been ethically reviewed by the Institutional Review Board of the Massachusetts Institute of Technology (MIT, Cambridge, Massachusetts, USA). The database protects patient privacy; thus, the requirement for informed consent was waived.

The extracted variables were as follows: age, sex, admission time, follow-up time, severity score, comorbidities, and laboratory parameters. Comorbidities included congestive heart failure, sepsis, renal disease, liver disease, pneumonia, diabetes mellitus, hypertension, and respiratory failure. Laboratory tests included leukocytes, hematocrit, red blood cell distribution width, hemoglobin, platelets, creatinine, glucose, blood urea nitrogen, prothrombin time, activated partial thromboplastin time, total serum bilirubin, and albumin. The data extracted in this experiment were obtained within 24 h of admission to the ICU.

### Inclusion and exclusion criteria

The criteria for inclusion in the Acute Kidney Injury Network (AKIN) were as follows: sudden (within 48 h) decline in renal function as evidenced by an absolute increase in serum creatinine ≥ 0.3 mg/dl (≥ 26.4 μmol/l), a percentage increase in serum creatinine ≥ 50% (1.5 times the baseline), or a decrease in urine output (< 0.5 ml/kg/h for more than 6 h) [17]. First, because the MIMICIII database contains data from 2002–2012, it predates the Kidney Disease Improving Global Outcomes (KDIGO) criteria (2012) release. Second, the systematic evaluation showed no significant differences in performance between the End-stage renal disease (RIFLE), AKIN, and KDIGO outcome definitions [18]. Finally, many studies on AKI also use AKIN [6, 19–27]. Therefore, the AKIN criteria were used in this study. Adult patients (≥18 years) diagnosed with AKI in the MIMIC-III database with both serum bilirubin and albumin data on record were included in this study.

Patients older than 18 years admitted for the first time were selected. The exclusion criteria were as follows: (1) patients not admitted for the first time; (2) ICU admission <48 h; (3) patients with missing total bilirubin and albumin data, and (4) pregnant women and oncology patients.

### Statistical analysis

#### The study population was divided into groups based on the dichotomous level of B/A on the first day of ICU stay

The primary outcome was 28-day all-cause mortality; the secondary outcomes were 60-day, 1-year, and 4-year all-cause mortality. Missing data in continuous variables were interpolated from nonmissing values, and covariates with ≥10% missing values were excluded. Normality tests were performed using the Kolmogorov–Smirnov method. Continuous data were described according to their data distribution, using the mean ± standard deviation for normally distributed data; for non-normality, the median was used. A *t*-test was used if the data involved continuous variables and were normally distributed between two groups; a non-parametric test was used for non-normality. Percentages were used for categorical data description; chi-squared analysis was used for tests.

Kaplan–Meier survival curves were constructed to compare all-cause mortality rates at 28 days, 60 days, 1 year and 4 years for both groups at the B/A level. Restricted cubic spline (RCS) regression models were used to show the relationship between B/A and HR at 28 days, 60 days, 1 year, and 4 years. Three multivariate Cox proportional risk models were constructed to determine the effect of B/A on mortality rates. Impact baseline variables were used as candidate predictors for the multivariate regression models, and variance inflation factor (VIF) was used to quantify multicollinearity between variables to prevent overfitting. Variables with VIF ≥10 were excluded. Patients were stratified by sex, age, and hypertension for the analysis to determine the effect of B/A on the primary outcome event.

The analyzes were performed with R software (version 4.2.2; R Foundation for Statistical Computing, Vienna, Austria) and SPSS statistical software (IBM SPSS Statistics, Version 26.0; Armonk, NY, USA). A two-sided P-value < 0.05 was considered statistically significant for all analyses.

## Results

### Baseline characteristics

**Fig 1:**
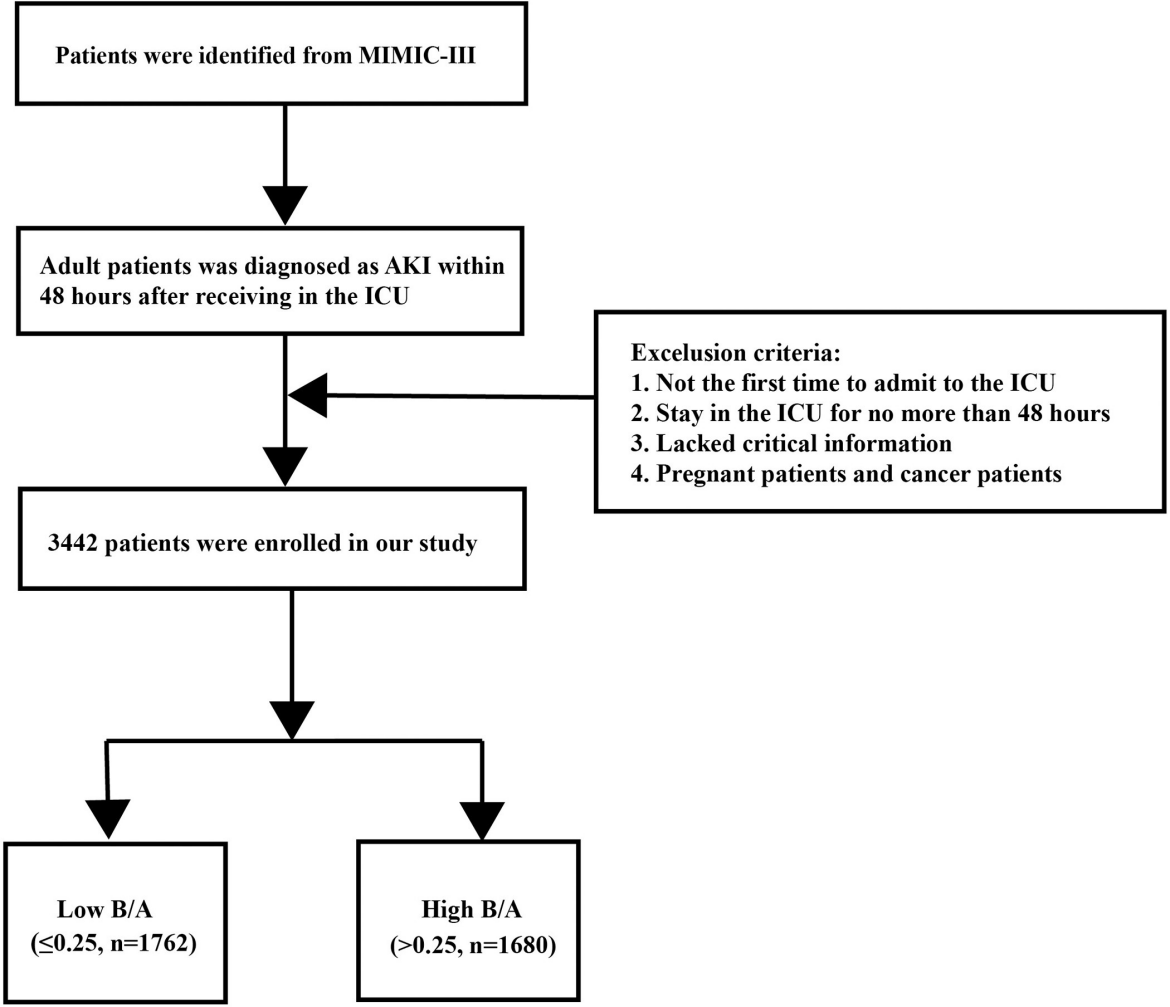
Flow chart of patient selection for the analysis.

The screening of study patients is shown in Fig 1 (Flowchart 1); 3,442 AKI patients with complete total bilirubin and albumin data obtained within 24 h of admission were finally included. A threshold value of 0.25 for B/A was derived on the basis of the statistical analysis of the B/A levels in two equal parts. The study population was divided into a high B/A group and a low B/A group. The baseline characteristics of the two groups are shown in Table 1. The two groups differed (P<0.05) in sex, age, score (SOFA, SAPSII, SAPS, and LODS), and comorbidities (diabetes, sepsis, congestive heart failure, hypertension, liver disease, and renal disease). There were differences in the incidence of liver disease, and sepsis rates were also higher in the high B/A group (both P<0.05).

**Table 1:** Comparison of the general characteristics between the two groups.

Categories	Low B/A	High B/A	P value
female, n (%)	806(45.70)	661(39.30)	<0.001
Age, years	67.10	62.69	<0.001
**Vital signs**			
Mean blood pressure, mmHg	56.00	55.00	0.002
Heartrate, beats/min	106.00	111.00	<0.001
Temperature, °C	37.60	37.61	0.62
**Score**			
SOFA,	5.00	8.00	<0.001
SAPSII	40.00	45.00	<0.001
SAPS	21.00	22.00	<0.001
LODS	5.00	6.00	<0.001
**Comorbidities, n (%)**			
Diabetes	597(33.90)	401(23.90)	<0.001
Sepsis-3	1248(70.80)	1402(83.50)	<0.001
Congestive heart failure	638(36.20)	494(29.40)	<0.001
Chronic-pulmonary	351(19.90)	273(16.30)	0.005
Liver disease	71(4.00)	419(24.94)	<0.001
Renal-failure	440(25.00)	269(16.00)	<0.001
Hypertension	1053(59.76)	753(44.80)	<0.001
**Laboratory parameters**			
Hemoglobin, g/dl	10.00	9.50	<0.001
Hematocrit, %	29.60	28.10	<0.001
RDW, %	14.70	15.40	<0.001
WBC, 10^9^/L	14.40	14.20	0.68
Bilirubin, mg/dl	0.4	1.7	<0.001
Albumin, g/dl	3.3	2.8	<0.001
Creatinine, mg/dl	1.60	1.70	0.08
Glucose, mg/dL	185	170	<0.001
APTT, s	34.60	41.50	<0.001
PT, s	14.60	17.70	<0.001
Uo-48h, mL	3405.00	2777.00	<0.001
Lactate, mmol/L	2.60	3.40	<0.001
**Mortality, n (%)**			
28-Day mortality	317(18.00)	421(25.10)	<0.001
60-Day mortality	380(21.60)	522(31.10)	<0.001
1-Year mortality	552(31.30)	670(39.90)	<0.001
4-Year mortality	745(42.30)	800(47.60)	0.002

APTT: Activated partial thromboplastin time; PT: Prothrombin time; WBC: White blood cell

RDW: Red blood cell distribution width; UO-48h:48h Urinary Output.

### Kaplan–Meier survival curve analysis

**Fig 2:**
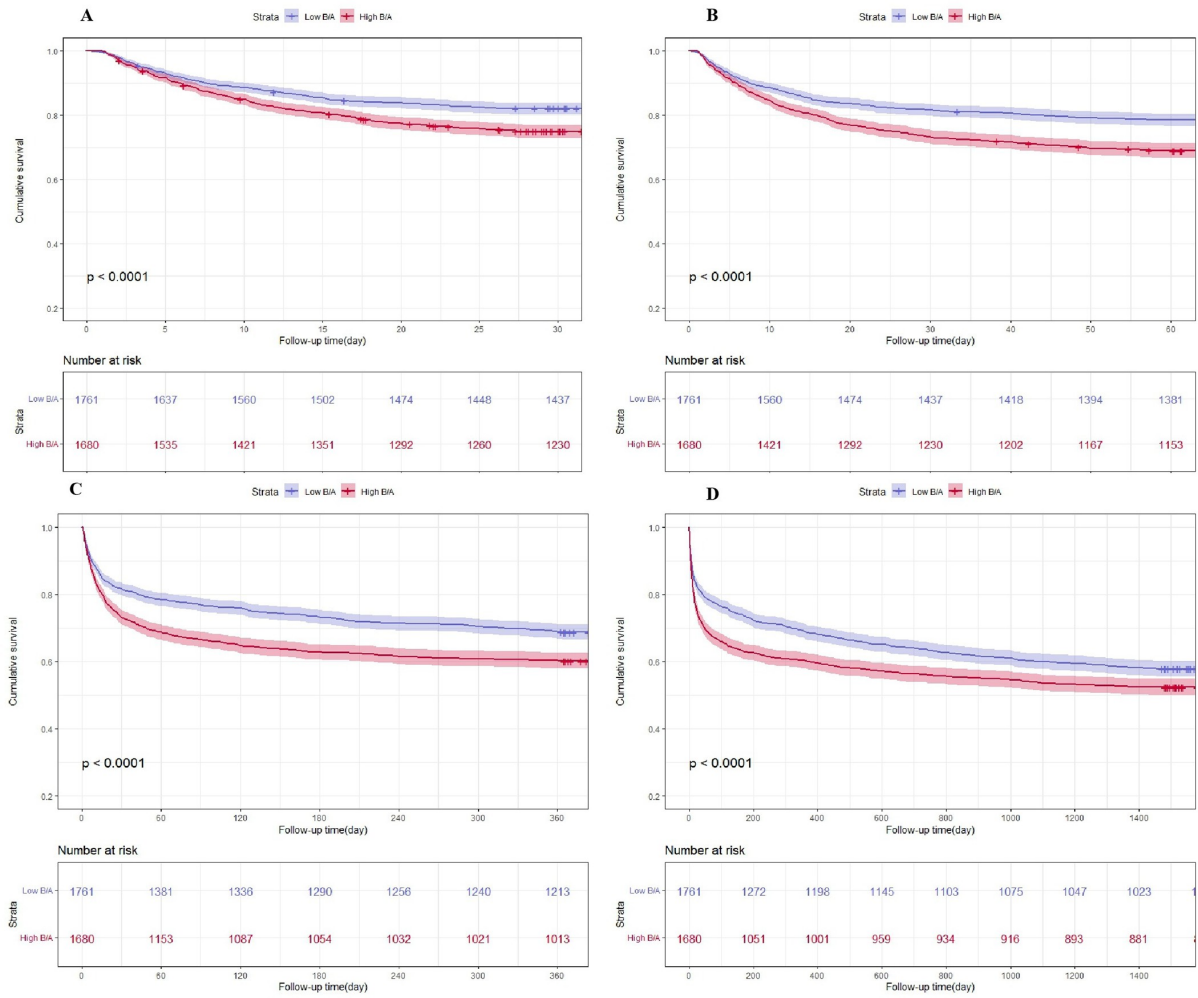
Kaplan–Meier survival analysis curves for all-cause mortality. B/A: Low B/A (≤0.25), High B/A (>0.25). Kaplan–Meier curves showing the cumulative probability of all-cause mortality in each group at 28 days (A), a landmark analysis at 60 days (B), a landmark analysis at 1 year (C), and the Kaplan–Meier survival analysis curves for all-cause mortality per group at 4 years (D).

Kaplan–Meier survival curves were plotted to represent the survival of AKI patients stratified into two groups by B/A levels at 28 days, 60 days, 1 year, and 4 years. The results showed that high B/A levels were associated with statistically significant differences in all-cause mortality at 28 days, 60 days, 1 year, and 4 years in patients with AKI (all P<0.0001) (Fig 2).

### The relationship between all-cause mortality and the B/A ratio

**Fig 3:**
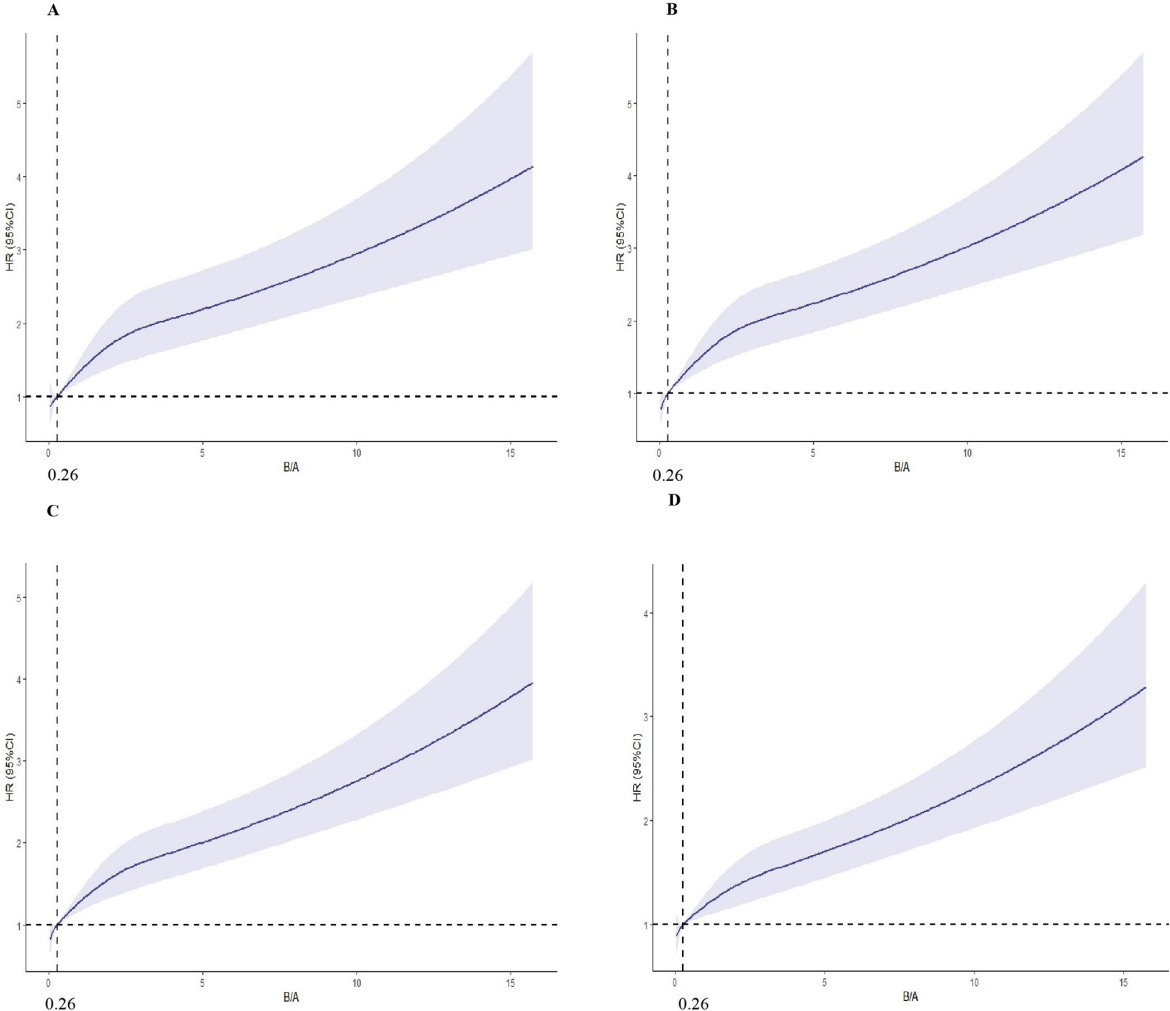
Restricted cubic spline regression analysis of B/A with all-cause mortality. Heavy central lines represent the estimated adjusted hazard ratios, with shaded ribbons denoting 95% confidence intervals. B/A 0.26 was selected as the reference level represented by the vertical dotted lines. The horizontal dotted lines represent the hazard ratio of 1.0. (A) Restricted cubic spline for 28-day mortality risk. (B) Restricted cubic spline for 60-day mortality risk. (C) Restricted cubic spline for 1-year mortality risk. (D) Restricted cubic spline for 4-year mortality risk. HR: hazard ratio, CI: confidence interval.

Three models were developed by unadjusted, fine-tuned, and fully adjusted Cox regression to further elucidate the relationship between B/A and the risk of death from AKI (Table 2). In the univariate Cox proportional risk regression, the risk ratios for B/A were 1.10 (95% CI, 1.07–1.12, P< 0.001), 1.10 (95% CI, 1.09–1.12, P<0.001),1.10 (95% CI, 1.08–1.11, P<0.001), and 1.08 (95% CI, 1.07–1.10, P<0.001), respectively. The multivariate-adjusted HR showed that B/A was an independent predictor of short-and long-term mortality in patients with AKI [28-day mortality (model 3: HR = 1.10, 95% CI = 1.08–1.13, P<0. 001)]. RCS regression models showed that high B/A was associated with an increased risk of mortality at 28 days, 60 days, 1 year, and 4 years (Fig 3).

**Table 2:** Cox proportional hazard ratios (HR) for all-cause mortality.

Outcomes	Model 1 HR (95%CI)	P value	Model 2 HR (95%CI)	P value	Model 3 HR (95%CI)	P value
28-day mortality						
B/A	1.10(1.08~1.12)	<0.001	1.11(1.09~1.12)	<0.001	1.10(1.08~1.13)	<0.001
Low B/A	Ref		Ref		Ref	
High B/A	1.45(1.25~1.68)	<0.001	1.50(1.30~1.74)	<0.001	1.24(1.05~1.45)	0.01
60-day mortality						
B/A	1.10(1.09~1.12)	<0.001	1.11(1.09~1.13)	<0.001	1.11(1.09~1.13)	<0.001
Low B/A	Ref		Ref		Ref	
High B/A	1.51(1.33~1.73)	<0.001	1.59(1.39~1.81)	<0.001	1.30(1.12~1.51)	<0.001
1-year mortality						
B/A	1.10(1.08~1.11)	<0.001	1.11(1.09~1.12)	<0.001	1.11(1.09~1.13)	
Low B/A	Ref		Ref		Ref	
High B/A	1.38(1.23~1.54)	<0.001	1.46(1.30~1.63)	<0.001	1.24(1.09~1.40)	0.001
4-year mortality						
B/A	1.08(1.07~1.10)	<0.001	1.10(1.09~1.12)	<0.001	1.09(1.07~1.11)	<0.001
Low B/A	Ref		Ref		Ref	
High B/A	1.23(1.11~1.36)	<0.001	1.31(1.18~1.44)	<0.001	1.15(1.03~1.28)	0.014

Model 1: Unadjusted

Model 2: Adjusted for age and sex

Model 3: Adjusted for age, sex, sepsis-3, congestive heart failure, acute respiratory distress syndrome, cardiac arrhythmias, valvular disease, pulmonary circulation, peripheral vascular, hypertension, renal failure, liver disease, white blood cell, mean blood pressure, hemoglobin, hematocrit, temperature, heart rate, creatinine, glucose, QSOFA, GCS, and SIRS.

**Fig 4:**
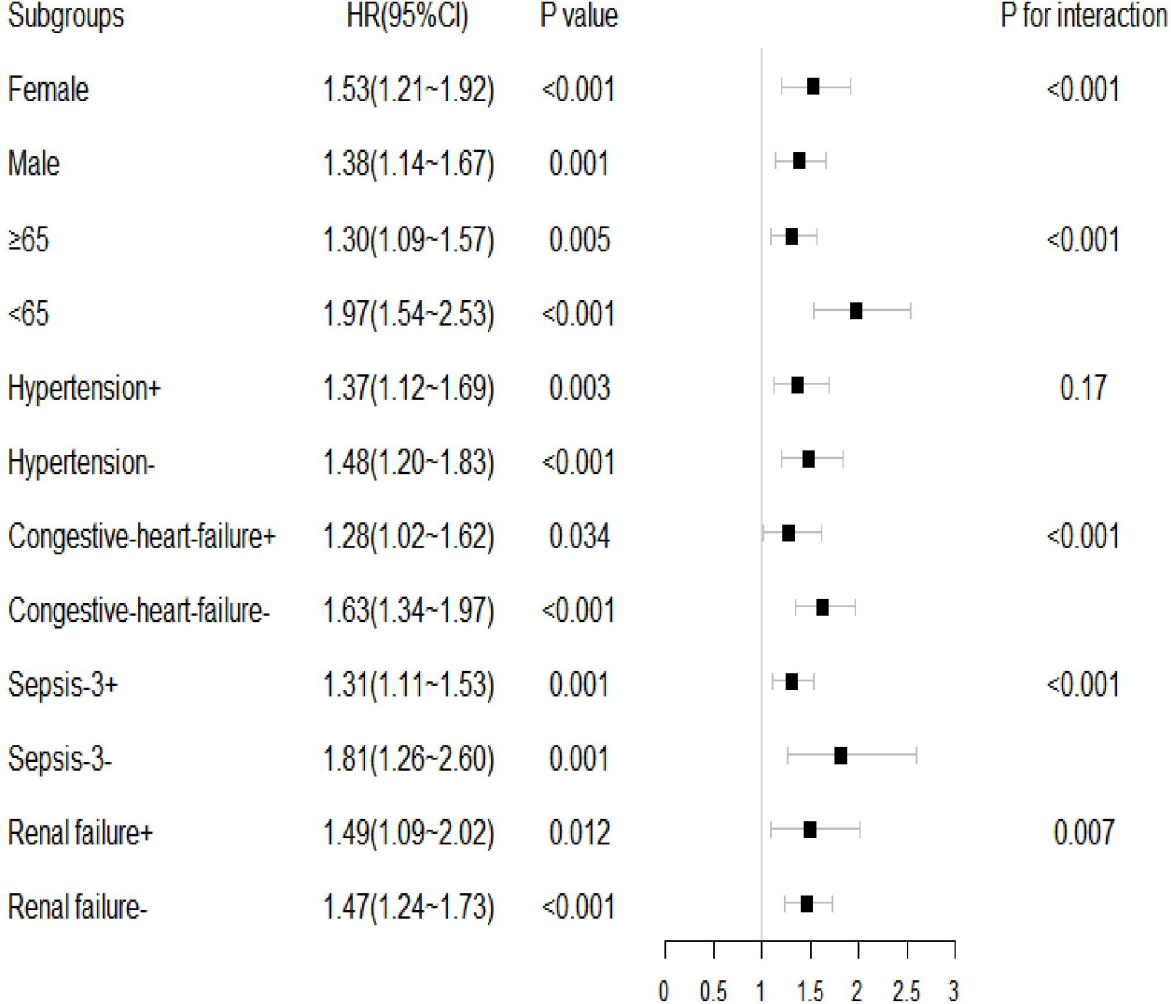
Forest plots of hazard ratios for the 28-day mortality risk in different subgroups. HR: hazard ratio, CI: confidence interval.

A stratified analysis of the relationship between B/A and mortality in AKI patients was performed according to potential factors such as sex, age, and hypertension. The results of the subgroup analysis showed (Fig 4) that sex, age, presence of hypertension, congestive heart failure, sepsis, and renal failure differed in terms of B/A-associated mortality risk at 28 days in both groups. However, there was no significant interaction in the hypertensive group (P for interaction = 0.17).

## Discussion

The analysis of the relationship between B/A and AKI patient prognosis revealed that patients in the high B/A group had significantly higher mortality rates at 28 days, 60 days, 1 year, and 4 years than those in the low B/A group. Then, after adjusting for age and sex, higher B/A was associated with increased all-cause mortality at 28 days, 60 days, 1 year, and 4 years in AKI patients. In addition, B/A was also an independent predictor of all-cause mortality in these patients after adjusting for more confounding factors. Thus, the study suggests that B/A is a predictor of short-and long-term mortality in patients with AKI. Both total bilirubin and albumin levels are readily available, and the B/A ratio can help assess the long-term prognosis of patients with AKI after hospital discharge.

High levels of total serum bilirubin are associated with liver dysfunction and lead to an imbalanced immune response, increased bacterial infection, and impaired renal or cardiac function, all of which are strongly associated with increased mortality risk [28]. Related studies have shown that serum total bilirubin concentrations are independently associated with in-hospital mortality in critically ill adults; patients with serum total bilirubin levels ≥ 2 mg/dL had an in-hospital mortality rate of 31.9% [29]. Risk Investigators et al. also showed that elevated serum total bilirubin levels were independently associated with AKI in hospitalized patients [30].

Albumin has various functions, such as carrying poorly water-soluble molecules, regulating osmotic pressure, antioxidant activity and anti-inflammatory effects, which have a protective effect on the kidney [31]. Hypoalbuminemia prevents the patient’s kidneys from effectively removing toxic substances and decreases vascular volume, affecting renal perfusion; both effects can cause kidney damage [32]. Studies have shown that patients with serum albumin levels ≤2.4 g/L have an increased risk for AKI [33]. Albumin infusion significantly increases renal blood flow, shifting the renal blood flow autoregulation curve toward normalization and improving renal function [34].

The serum total bilirubin to albumin ratio is also important in disease prediction. Hyperbilirubinemia with hypoalbuminemia occurs frequently and is associated with poor prognosis in critically ill patients; Choi JS et al. [16] showed that the B/A ratio was significantly associated with 28-day mortality in ICU patients using a multivariate analysis adjusted for age, sex, and underlying disease. The survival analysis showed that a higher B/A ratio was associated with poor prognosis and mortality in critically ill patients. In the current study, the survival analysis results also showed that a high B/A ratio is associated with mortality in patients with AKI. Previous studies have shown that the B/A ratio plays an important role in predicting acute bilirubin-induced neurological dysfunction, with patients with acute bilirubin-induced neurological dysfunction demonstrating a significantly higher B/A ratio than those without (P<0.001) [35]. A correlation between B/A ratio levels and bilirubin encephalopathy has been shown, with higher B/A ratios increasing the bilirubin encephalopathy risk by 23% [36]. In addition, a high B/A ratio can predict resistance to intravenous immunoglobulin in patients at high risk for Kawasaki disease [37].

Although several scoring systems are available for predicting mortality in critically ill patients, using the B/A ratio is advantageous because it is easy to measure [16]. However, our study has limitations. As this was a retrospective study, causality could not be determined. The Medical Information Marketplace critical care database is a large single-center database and lacks diversity. Therefore, prospective studies are needed to confirm our findings.

## Conclusions

The current study of clinical data from 3442 AKI patients in the MIMIC-III database revealed that higher B/A ratios among AKI patients were associated with increased all-cause mortality at 28 days, 60 days, 1 year, and 4 years. The B/A ratio was found to be a risk factor for prognosis in AKI patients. Therefore, clinicians can use B/A values to predict clinical outcomes in AKI patients and help improve patient prognosis.
